# Dynamic nature of somatic chromosomal mosaicism, genetic-environmental interactions and therapeutic opportunities in disease and aging

**DOI:** 10.1186/s13039-020-00488-0

**Published:** 2020-05-07

**Authors:** Svetlana G. Vorsanova, Yuri B. Yurov, Ivan Y. Iourov

**Affiliations:** 1 Veltischev Research and Clinical Institute for Pediatrics of the Pirogov Russian National Research Medical University, Ministry of Health of Russian Federation, 125412 Moscow, Russia; 2 Mental Health Research Center, 117152 Moscow, Russia; 3 Department of Medical Biological Disciplines, Belgorod State University, 308015 Belgorod, Russia

**Keywords:** Aneuploidy, Chromosome, Disease, Environment, Ontogeny, Somatic mosaicism, Therapy

## Abstract

**Background:**

Somatic chromosomal mosaicism is the presence of cell populations differing with respect to the chromosome complements (e.g. normal and abnormal) in an individual. Chromosomal mosaicism is associated with a wide spectrum of disease conditions and aging. Studying somatic genome variations has indicated that amounts of chromosomally abnormal cells are likely to be unstable. As a result, dynamic changes of mosaicism rates occur through ontogeny. Additionally, a correlation between disease severity and mosaicism rates appears to exist. High mosaicism rates are usually associated with severe disease phenotypes, whereas low-level mosaicism is generally observed in milder disease phenotypes or in presumably unaffected individuals. Here, we hypothesize that dynamic nature of somatic chromosomal mosaicism may result from genetic-environmental interactions creating therapeutic opportunities in the associated diseases and aging.

**Conclusion:**

Genetic-environmental interactions seem to contribute to the dynamic nature of somatic mosaicism. Accordingly, an external influence on cellular populations may shift the ratio of karyotypically normal and abnormal cells in favor of an increase in the amount of cells without chromosome rearrangements. Taking into account the role of somatic chromosomal mosaicism in health and disease, we have hypothesized that artificial changing of somatic mosaicism rates may be beneficial in individuals suffering from the associated diseases and/or behavioral or reproductive problems. In addition, such therapeutic procedures might be useful for anti-aging strategies (i.e. possible rejuvenation through a decrease in levels of chromosomal mosaicism) increasing the lifespan. Finally, the hypothesis appears to be applicable to any type of somatic mosacism.

*Πάντα χωρεῖ καὶ οὐὲν μένει*

*(Everything flows and nothing stays)*

*Heraclitus of Ephesus*

Somatic chromosomal mosaicism is the presence of chromosomally distinct cellular populations in an individual. This type of intercellular genomic variations is commonly associated with a wide spectrum of genetic diseases ranging from chromosomal syndromes to complex disorders. Furthermore, somatic chromosomal mosaicism is a risk factor for cancer and reproductive problems [1–7]. Increases and decreases in numbers of cells with abnormal karyoptypes are systematically observed in humans through ontogeny (i.e. from zygote to death). The dynamic fluctuations in mosaicism rates have been suggested to be a mechanism for intrauterine control of cell numbers and for aging [8–11]. It is noteworthy that mosaicism rates may change due to genetic-environmental interactions [12, 13]. Alternatively, mosaic individuals may demonstrate a reversion to normal of inherited mutations [14, 15]. Moreover, a number of techniques for artificial changing of mosaicism levels appear to exist (e.g. CRISPR/Cas9-mediated genome editing) [16]. Additionally, there is a line of evidences for a kind of self-correction of chromosome abnormalities (decrease of mosaicism rates) in early mammalian development [17–19]. Taking into account these features of somatic mosaicism, we have hypothesized that dynamic changes in rates of chromosomal mosaicism mediated by genetic-environmental interactions are able to deliver therapeutic opportunities in disease and aging.

Almost all types of chromosomal abnormalities (aneuploidy/polyploidy, structural rearrangements, supernumerary marker chromosomes) are able to be mosaic [7, 20–23]. Despite the formation mechanisms (i.e. zygotic or self-correction of chromosomal abnormalities versus post-zygotic or somatic mutagenesis), mosaic chromosomal abnormalities are generally associated with reduced phenotypic penetrance and decreased stability of cellular genomes as compared to regular/non-mosaic ones [3, 7, 10–13, 24, 25]. Somatic chromosomal mosaicism is common in clinical cohorts of patients with neurodevelopmental disabilities and/or congenital anomalies [7, 25–27]. Because of ontogenetic (“ontogenomic”) variations, mosaic genome variations causing a wide spectrum of disorders require specific approaches to the diagnosis and management including molecular cytogenetic monitoring of ontogenetic changes in mosaicism rates [28]. Actually, a large amount of data acquired through cytogenetic analyses of mosaicism over the last 50 years has indicated less severe phenotypes of chromosomal disorders to be associated with mosaicism, the rates of which are likely to change ontogenetically.

In early ontogeny, somatic variations of the human genome seem to achieve unprecedently high rates (i.e. the amount of chromosomally abnormal cells achieves the “ontogenetic” maximum). The latter stages are associated with a decrease in chromosomal instability (mosaicism) rates, which still remain high [9, 10, 29, 30]. In parallel, chromosomal mosaicism/instability confines to either extraembryonic or embryonic tissues (i.e. chromosomal mosaicism confined to placenta or fetal brain, respectively) [31–34]. The consequences of these intercellular genomic variations may be devastative at later developmental stages [35, 36]. For instance, high rates of chromosomal mosaicism are associated with ~ 1/4 of spontaneous abortions in the first trimester [37–39]. On the other hand, lower rates of chromosomal mosaicism and instability are able to contribute to postnatal morbidity being causative per se or being an element of pathogenic cascades in complex diseases [3, 7, 40]. In the latter scenario (i.e. mosaicism is an element of a pathogenic cascade), chromosomal mosaicism and instability are more likely to result from altered programmed cell death and/or failed cellular selection/clearance through gestation [9, 10, 33–35]. These alterations seem to be common mechanisms for complex diseases mediated by genetic (chromosomal/genomic) instabilities.

There is a strong evidence that somatic chromosomal mosaicism and instability contributes to the pathogenesis of brain diseases [21, 41–45]. Chromosomal mosaicism has been systematically observed in autistic individuals [5, 7, 46, 47]. In schizophrenia, the diseased brain exhibits tissue-specific mosaicism manifested as aneuploidy and specific copy number variations [48–52]. Neurodegeneration has been found to be mediated by somatic aneuploidy and chromosomal instability confined to degenerating brain areas [44, 53–55]. More precisely, Alzheimer’s disease has been associated with brain-specific genomic/chromosomal instability (e.g. aneuploidy), which is integrated into the pathogenetic cascade of this devastating disease [56–62]. In addition, molecular (neuro)cytogenetic analyses of this late onset neurodegenerative disease have demonstrated that pathological aging is likely to be mediated by mosaic aneuploidy and chromosome instability in humans [63–65]. For more details, see reviews: [12, 21, 41–43, 45, 66]. Behavioral variability and post-traumatic stress disorders are likely to be mediated by genomic/chromosomal instability and somatic mosaicism, as well [67, 68]. Here, it is important to note that changes of cell proportions are hypothesized to determine the dynamics of behavioral variability (i.e. an increase of abnormal cell numbers may lead to more severe behavioral problems, whereas a decrease of abnormal cell numbers is likely to diminish the severity of behavioral problems) [68]. Finally, reproductive problems have long been associated with chromosomal mosaicism, affecting either fetuses or individuals experiencing reproductive failure [4, 9, 20, 22, 35–39]. In total, studying brain diseases in the context of somatic mosaicism suggests that intercellular genetic heterogeneity (chromosomal heterogeneity) is a mechanism for central nervous system dysfunction and the dynamic nature determines the phenotypic outcome. Additionally, empirical and theoretical observations show that a correlation between changes in mosaicism levels and phenotypic manifestations does exist.

Another picturesque example of somatic mosaicism’s impact on human homeostasis is aging. Dynamic changes of mosaicism rates produced by the accumulation of somatic mutations (i.e. aneuploidy) seem to be an important cytogenetic mechanism for human aging [69–71]. Cytogenetic and cytogenomic studies of normal and pathological aging consistently demonstrate an increase in rates of chromosomal mosaicism and instability in relation to age [10, 55, 63, 64, 70]. Since 60s, the latest ontogenetic stages have been associated with higher rates of chromosomal mosaicism and instability [7, 8, 11]. Thus, these data allow to hypothesize that external inhibition of age-dependent chromosome instability and a decrease of somatic chromosomal mosaicism rates might be an opportunity for anti-aging therapeutic interventions [10, 70]. Furthermore, somatic cancer-associated mutations commonly occur in aged human tissues of presumably healthy individuals [72]. It is not surprising inasmuch as chromosomal mosaicism and instabilities are risk factors for cancers [73, 74]. In general, aging-related diseases are commonly mediated by chromosomal instability and/or mosaic aneuploidy [7, 41, 44, 45, 55, 75–77]. The results of molecular genetic studies of aging correlate with observations on mutation load contribution to limiting/shortening the lifespan [78, 79]. Additionally, there are evidences that inhibiting chromosome instability might underlie successful anti-aging strategies [80]. Thus, genetic instability at chromosomal level involved in human aging and/or lifespan shortening is an intriguing target for lifespan-extension and anti-aging interventions.

Genetic-environmental interactions play an important role in generating chromosome instability and, probably, somatic chromosomal mosaicism [12, 13, 44]. It is highly likely that environmental triggers are able to stimulate or to inhibit genome/chromosome instability [7, 12, 13]. Here, it is to mention that a cellular genome may demonstrate a kind of a self-correction resulting in a corrected/normal genomes in daughter cells [14, 15, 17, 18]. Consequently, one can suggest the cellular genome has high self-correctional potential. Alternatively, somatic mosaicism is able to be a stress response or cellular adaptation to adverse environmental effects [13, 40]. Moreover, actual technologies of in vivo correction of cellular genomes have the intrinsic potential for becoming more safe and efficient in forthcoming future [16, 80]. Therefore, either special genome editing technologies (e.g. CRISPR/Cas9-mediated methods) or stimulated genetic-environmental interactions (i.e. medication, life style, diet, (anti-)stress etc.) are able to decrease levels of chromosomal mosaicism/instability. According to our hypothesis, these opportunities might be used for decreasing the risks for complex diseases/conditions, improving the dynamics of genetic diseases caused by mosaicism, increasing the lifespan, and rejuvenating. Disease progression in cancers and neurodegenerative diseases is able to be slowed down by therapeutic interventions decreasing the levels of chromosomal mosaicism/instability. Similarly, such interventions could decrease the risk for complex diseases, cancer, reproductive and behavioral problems. Figure 1 illustrates schematically the outcome of such interventions.

**Fig. 1:**
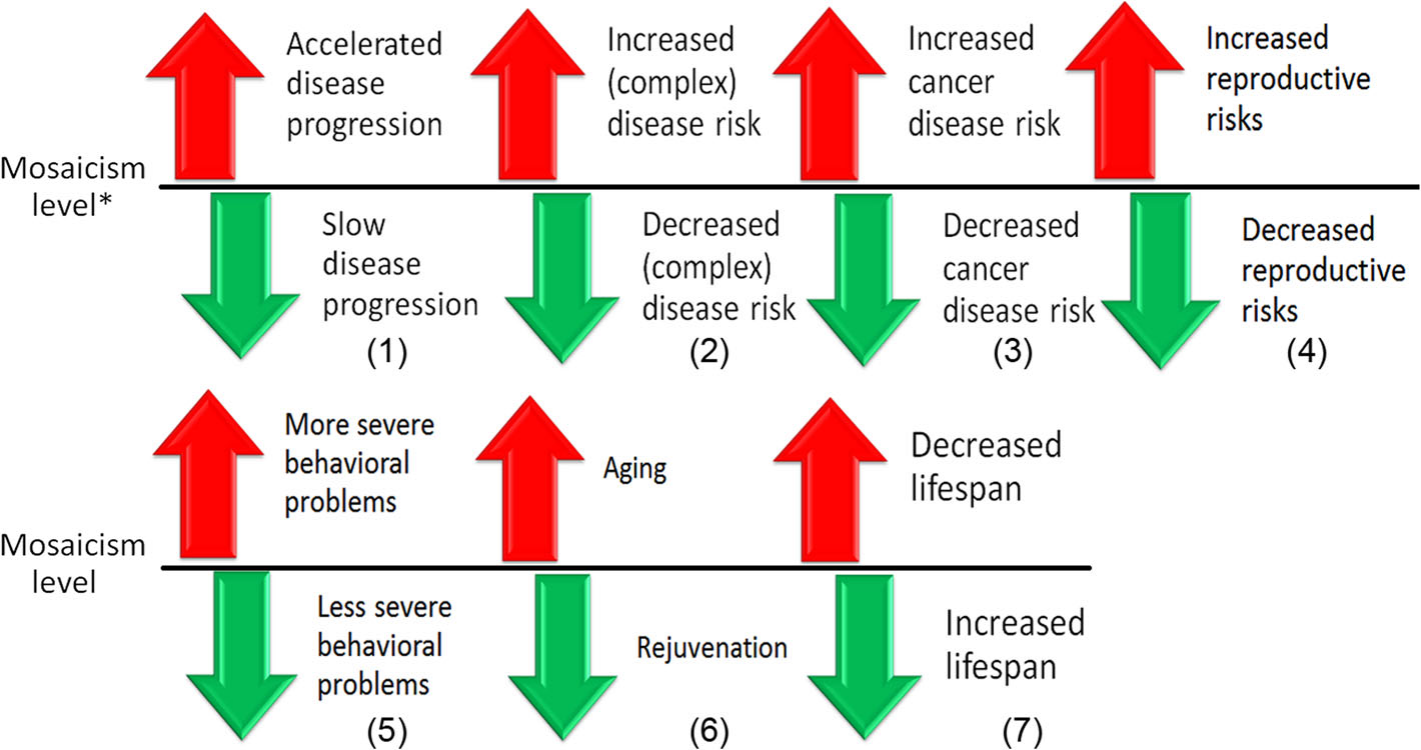
Biomedical issues of the hypothesis. When mosaicism is detected, therapeutic interventions might be applied to decrease the level of the mosaicism (green arrows). Otherwise, the level of mosaicism is more likely to increase (red arrows) through the interaction of unstable cellular genomes with the environment. Thus, a disease associated with somatic (chromosomal) mosaicism would exhibit accelerated progression without the interventions (i.e. the increase of mosaicism level) in contrast to slow progression resulted from therapeutic interventions aimed at the decrease in mosaicism level (1). Somatic mosaicism may be an important element of pathogenic cascade in complex diseases; the increase of mosaicism level is likely to increase the risk for these diseases, whereas the decrease of mosaicism level is likely to decrease the risk for these diseases (2). Similarly, the risk of cancer (3) and reproductive risks (4) might correlate with changing of mosaicism levels. According to a previous cytogenomic hypothesis [68], the severity of behavioral problems is able to be modulated by changing in levels of somatic mosaicism as depicted in (5). Since aging is mediated by the accumulation of somatic mutation (i.e. the increase of mosaicism level) [63, 69–74], it is highly likely that therapeutic interventions aimed at the decrease of mosaicism level might cause rejuvenation (6). Finally, taking into account (1–6), we hypothesize that the increase of mosaicism level is associated with decreased lifespan whereas the interventions aimed at the decrease of mosaicism level might increase the lifespan. * — mosaicism level detected in an individual during molecular (cytogenetic) analysis

In the postgenomic era, cytogenomic/cytogenetic analysis is required to uncover complemented molecular and cellular pathways to a disease and therapeutic interventions. Chromosome-oriented postgenomic studies are able to provide new understanding how genomic variations produce the phenotype at saupramolecular or nuclear level and what can be done to diminish the effect of causative mutations. The latter may be achieved by either correcting the pathways altered by chromosome abnormalities/instability or decreasing the number of cells carrying the mutations [81–84]. Since cancers are one of the most intriguing models for somatic mutagenesis, a number of the theoretical and empirical (oncocytogenetic) observations may contribute to our hypothesis. Taking into account that both clonal and nonclonal chromosomal aberrations (mosaic chromosome aberrations) are involved in cancers, changes in mosaicism rates for decreasing cancer risks (as suggested in Fig. 1.3) might be complicated [84, 85]. Depending on cancer phase, specific strategies for decreasing mosacism rates are to be developed. Furthermore, mosaicism has recently been suggested to be beneficial in some cancer cell populations (“trade-off” of cellular adaptation) [86]. In this instance, there is a need to develop approaches to differ between “beneficial” and “non-beneficial” mosaicism in cancers. In general, it is to conclude that therapeutic strategies to manage mosaicism rates should be personalized.

The results of studying the dynamic nature of somatic mosaicism and genetic-environmental interactions are relevant to a wide spectrum of biomedical fields (Fig. 1). The development of efficient procedures providing the decrease in levels of somatic genetic instability (chromosomal mosaicism/instability) would certainly be a breakthrough in modern biomedical science. To this end, it appears that our hypothesis is applicable to all the types of somatic mosaicism.

## Data Availability

not applicable.
